# Genetic Vulnerability and the Relationship of Commercial Germplasms of Maize in Brazil with the Nested Association Mapping Parents

**DOI:** 10.1371/journal.pone.0163739

**Published:** 2016-10-25

**Authors:** Luciano Rogério Braatz de Andrade, Roberto Fritsche Neto, Ítalo Stefanine Correia Granato, Gustavo César Sant’Ana, Pedro Patric Pinho Morais, Aluízio Borém

**Affiliations:** 1 Plants Science Department, Federal University of Viçosa, Viçosa, Minas Gerais, Brazil; 2 Genetics Department, Luiz de Queiroz College of Agriculture, University of São Paulo, Piracicaba, São Paulo, Brazil; 3 Systèmes biologiques, Centre de Coopération Internationale en Recherche Agronomique pour le Développement, Montpellier, Languedoc-Roussillo, France; USDA/ARS, UNITED STATES

## Abstract

A few breeding companies dominate the maize (Zea mays L.) hybrid market in Brazil: Monsanto^*®*^ (35%), DuPont Pioneer^*®*^ (30%), Dow Agrosciences^*®*^ (15%), Syngenta^*®*^ (10%) and Helix Sementes (4%). Therefore, it is important to monitor the genetic diversity in commercial germplasms as breeding practices, registration and marketing of new cultivars can lead to a significant reduction of the genetic diversity. Reduced genetic variation may lead to crop vulnerabilities, food insecurity and limited genetic gains following selection. The aim of this study was to evaluate the genetic vulnerability risk by examining the relationship between the commercial Brazilian maize germplasms and the Nested Association Mapping (NAM) Parents. For this purpose, we used the commercial hybrids with the largest market share in Brazil and the NAM parents. The hybrids were genotyped for 768 single nucleotide polymorphisms (SNPs), using the Illumina Goldengate^*®*^ platform. The NAM parent genomic data, comprising 1,536 SNPs for each line, were obtained from the Panzea data bank. The population structure, genetic diversity and the correlation between allele frequencies were analyzed. Based on the estimated effective population size and genetic variability, it was found that there is a low risk of genetic vulnerability in the commercial Brazilian maize germplasms. However, the genetic diversity is lower than those found in the NAM parents. Furthermore, the Brazilian germplasms presented no close relations with most NAM parents, except B73. This indicates that B73, or its heterotic group (Iowa Stiff Stalk Synthetic), contributed to the development of the commercial Brazilian germplasms.

## Introduction

The maize crop has drastically changed with the adoption of hybrid cultivars, in 1933 in the United States, 1944 in Brazil [1] and 1950 in Europe [2]. According to Duvick [3], the use of hybrid cultivars is beneficial due to increased yields, stability and uniformity of the crops. Increased yields directly influence farmers’ profits, stability increases crop tolerances to both abiotic and biotic stresses, and uniformity facilitates agricultural mechanization and thereby increases cultivable area. However, production based on hybrid cultivars reduces the genetic pool of commercial germplasms [4]. This is evidenced by the fact that the main maize hybrid cultivars in the world come from crosses involving only seven lines: B73, Mo17, LH82, LH123, PH207, PH59 and PHG39 [4].

According to Romay et al. [5], the limited genetic variability of the current commercial maize germplasms may lead to increased vulnerability to abiotic and biotic stresses and, thus, result in food insecurity and limited gains from crop selection. The vulnerability is most evident when the crop is present in large areas and in monoculture systems, in general, of a single or of only a few cultivars [6]. In this context, the maize crop has been considered to be at risk for genetic vulnerability. One example of this vulnerability was the Northern leaf blight (*Exserohilum turcicum*) epidemic in 1970, which elicited concern regarding the genetic diversity among American breeder hybrids [7].

Currently, the maize crop in Brazil makes use of improved seeds in 14 million hectares [8], in addition the majority of the varieties are hybrid cultivars (88.32%) [9]. This market is dominated by the following companies: Monsanto^®^, 35% of the market-share, followed by Dupont Pioneer^®^ (30%), Dow Agrosciences^®^ (15%), Syngenta^®^ (10%), Helix Sementes^®^ (4%), EMBRAPA (3%) and other companies (3%). According to Reif et al. [10], monitoring genetic diversity is important in such a scenario because the breeding practices, registration and the marketing of new cultivars may lead to increased genetic vulnerability.

Genetic diversity is mainly estimated through genotypic information. Due to the large increase in characterized markers and automated sequencing platforms available, the use of single nucleotide polymorphisms (SNPs) has been increasing. These markers are biallelic and have good coverage across the maize genome [11], which enables their application in studies of genetic diversity and population structure [12] as well as in examining the relationships and origins of germplasms [13,14].

Thus, aiming to provide genetic resources for the study of quantitative traits and diversity in maize, McMullen et al. [15] developed the Nested Association Mapping (NAM) population. Therefore, the selection of the NAM parents aimed to represent the global maize genetic diversity, and to be divergent from the B73 line [15,16].

Considering all of the factors above, the objectives of this study were to estimate the risk of genetic vulnerability in the Brazilian commercial maize germplasms and the genetic kinship of these germplasms with the NAM parents.

## Materials and Methods

### Genetic Backgrounds

Maize hybrids of the leading Brazilian market share companies were used to represent the commercial Brazilian maize germplasm. Monsanto^®^ was represented by the Dekalb^®^ brand, while Dow Agrosciences^®^ and Agromen Tecnologia^®^ were self-represented. Similarly, Helix Sementes^®^ was represented by the brands Biomatrix^®^ and Santa Helena Sementes^®^. These hybrids (Population one) display high adaptability, and the majority of them are transgenic organisms (Table 1).

**Table 1:** Description of 20 commercial maize hybrids used in this study, Population one.

Company	Hybrid	Type	Cycle	Type of Corn	Crop Purpose
Agromen Tecnologia	30A37PW^a^	SC	Early	Semi Dent	Grain
Biomatrix	BM820	SC	Early	Flint	Grain
	BM207	DC	Early	Semi Flint	Grain and Silage
	BM915PRO^b^	SC	Super Young	Semi Dent	Grain and Silage
Dekalb	DKB 340 PRO^b^	SC	Semi early	Semi Flint	Grain
	DKB 310 PRO^b^	SC	Regular	Semi Flint	Grain
	DKB 390	SC	Early	Flint	Grain
	DKB 177 PRO^b^	SC	Early	Semi Flint	Grain
Dow Agrosciences	2B688PW^a^	TW	Early	Semi Flint	Grain and Silage
	2B587PW^a^	SC	Early	Semi Dent	Grain
	2B710PW^a^	SC	Early	Semi Flint	Grain
	2B810PW^a^	SC	Regular	Semi Flint	Grain
	2A550PW^a^	SC	Early	Semi Flint	Grain
DuPont Pioneer	P4285H^c^	SC	Early	Flint	Grain and Silage
	30F53H^c^	SC	Early	Semi Flint	Grain and Silage
Santa Helena	SSC 5560	TW	Early	Flint	Grain and Silage
Syngenta	Truck TL^d^	SC'	Early	Flint	Grain
	Fórmula TL^d^	SC	Super young	Flint	Grain
	Status Viptera^e^	SC	Early	Flint	Grain
	Impacto TL^d^	SC	Early	Flint	Grain

SC: Single cross, SC’: Modified single cross, DC: Double cross, TW: Three way. Superscript letters represent the transgenic technology used in hybrid:

^a^ PowerCore^®^,

^b^ YieldGard^®^,

^c^ Herculex^®^,

^d^ TL^®^,

^e^ Viptera^®^

Aiming to learn the main sources of the current Brazilian germplasm, it was included along with previously described hybrids, the NAM parental genotypes (www.panzea.org) and the Mo17 inbred line [15] (Population two; Table 2). The Mo17 inbred line was used due to it is importance in maize breeding and its higher specific combination capacity with the B73 inbred line.

**Table 2:** Parents of NAM used to discover the possible origins of Brazilian maize germoplasms, Population two.

Inbred Line	Group/Origin	Inbred Line	Group/Origin
B73	Stiff stalk	Ky21	Non-stiff stalk
B97	Non-stiff stalk	M162W	Non-stiff-stalk
CML103	Tropical-Subtropical	M37W	-
CML228	Tropical-subtropical	Mo17	Lancaster
CML247	Tropical-subtropical	Mo18W	-
CML277	Tropical-subtropical	MS71	Non-stiff stalk
CML322	Tropical-subtropical	NC350	Tropical-subtropical
CML333	Tropical-subtropical	NC358	Tropical-subtropical
CML52	Tropical-subtropical	Oh43	Lancaster
CML69	Tropical-subtropical	Oh7b	-
Hp301	Popcorn	P39	Sweet corn
I114H	Sweet corn	Tx303	-
Ki3	Tropical-subtropical	Tzi8	Tropical-subtropical
Ki11	Tropical-subtropical		

Source: Liu et al. [16]

### Genotyping and quality control procedures

Samples of leaf tissue from each hybrid were sent to the DuPont Pioneer^®^ Company, where DNA extraction and genotyping was performed. The latter was performed using the Illumina GoldenGate^®^ Platform (Illumina, San Diego, CA, USA), with an array of 768 SNPs [17]. The SNP panel was developed by Nelson et al. [18] to make inferences about the population structure, origins and relationships between American commercial hybrid parents with Plant Variety Protection Act (PVPA) expired.

The NAM parental lines were genotyped using an array of 1,536 SNP markers on the Illumina GoldenGate^®^ platform, and the data are available at www.panzea.org. Using the NAM genotype data, only the SNPs shared with the Brazilian commercial germplasms were analyzed.

The SNP genotypic data were converted into numeric digits (“1”, “2” and “3”) by means of the Scrime package [19] of the R program [20] (Vienna, Austria). Quality control of the genomic data was performed using the parameters of call rate (90%) and minor frequency allele (MAF, 5%) using the HapEstXXR package [21] of the R program.

### Data analysis

For each SNP marker, estimates were generated among the Brazilian commercial hybrid population and for the combined population (hybrids and NAM parents) by means of PowerMarker 3.25 software [22]:

1. Gene Diversity—*D*_*l*_
  $$D_{l}=1-\overset{k}{\underset{i=1}{\sum }}p_{li}^{2}$$ (1)
  in which *p*_*li*_ is the frequency of the *i-th* allele of the *l-th* SNP.
2. Minor Allele Frequency—MAF
  $$MAF_{l}=\frac{Q_{l}+R_{l}}{2\times \left(P_{l}+Q_{l}+R_{l}\right)}$$ (2)
  in which *Q*_*l*_ is the sum of heterozygous loci at the *l-th* SNP, *P*_*l*_ is the sum of homozygous loci for the *i-th* allele at the *l-th* SNP, and *R*_*l*_ is the sum of homozygous loci for the alternative, lower frequency *i-th* allele at the *l-th* SNP.
3. Heterozygosity—*H*_*l*_
  $$H_{l}=\frac{Q_{l}}{P_{l}+Q_{l}+R_{l}}$$ (3)
  in which *Q*_*l*_ is the sum of heterozygous loci at the *l-th* SNP, *P*_*l*_ is the sum of homozygous loci for the *ith-* allele at the *l-th* SNP, and *R*_*l*_ is the sum of homozygous loci for the *i-th*, lower frequency allele at the *l-th* SNP.
4. Polymorphism Information Content—PIC
  $$PIC_{l}=1-\overset{k}{\underset{i=1}{\sum }}p_{li}^{2}-\overset{k-1}{\underset{i=1}{\sum }}\overset{k}{\underset{j=i+1}{\sum }}2p_{li}^{2}p_{lj}^{2}$$ (4)
  in which *p*_*li*_ and *p*_*lj*_ are the frequency of the *i-th* and *j-th* alleles, respectively, at the *l-th* SNP.

From the allelic frequencies we estimated the genetic distances as described by Nei et al. [23]:

$$D_{A}=\frac{1}{r}\overset{r}{\underset{j=1}{\sum }}\left(1-\overset{m_{j}}{\underset{i=1}{\sum }}\sqrt{x_{ij}y_{ij}}\right)$$ (5)

in which *x*_*ij*_ and *y*_*ij*_ are the frequencies of the *i-th* allele at the *l-th* SNP on X and Y populations, respectively. *r* is the number of loci studied.

Subsequently, a dendrogram was generated using a bootstrap method (2,000 iterations), using the unweighted pair group method average (UPGMA). These bootstrap analyses were performed using the PowerMarker 3.25 software, and the node consistency dendrogram was generated using the MEGA 5.1 software [24].

The estimates of effective population size (*N*_*e*_) of the Brazilian commercial hybrids was obtained using the following equation:

$$N_{e}=\frac{1}{1+F_{st}}$$ (6)

and the *N*_*e*_ estimate of the hybrid progenitors was obtained using:

$$N_{e}=\frac{1}{2F_{st}}$$ (7)

in which *F*_*st*_ is the withinpopulation- inbreeding coefficient of the commercial germplasm. The latter was estimated as described by Resende et al. [25] using the genomic kinship matrix (G). *G* is estimated by:

$$G=\frac{WW\prime }{\overset{n}{\underset{i=1}{\sum }}2p_{i}q_{i}}$$ (8)

in which *W* is the incidence matrix to fix effects of alleles at bialellic markers, and *p*_*i*_ and *q*_*i*_ are allelic frequencies of biallelic markers at the *i-th* loci. So *F*_*st*_ was obtained by the mean of the diagonal of the G matrix less one.

The identification of private alleles of each company was performed with the Convert 1.31 software [26]. Private alleles of a given locus were considered to be those present only in the given population and absent in all others. The positions of the private alleles and available information regarding the associated metabolic and biologic functions of the respective genes were recorded using the Gramene platform (www.gramene.org) [27].

To quantify the kinship between the germplasms of the market share companies, Pearson correlation analysis was performed between the companies using the allele frequencies of the SNPs.

Aiming to identify the population structure of the elite Brazilian germplasms of maize breeding companies (Population one) and their relationship with NAM parents (Population two), two population structure analyses were performed using the Structure 2.3.4 Software [28]. In the first, only the genotypes of population one were considered, while in the second the genotypes of both Populations one and two were considered. The following parameters were used in generating the structures: admixture was modelled between the ancestral populations, with the number of ancestral populations (*K*) ranging from 1 to 10, with 10 repetitions for each K. For each run, the Markov Chain Monte Carlo (MCMC) number was one million, with burn-in of the first 500 thousand MCMC.

To estimate the number of ancestral populations that best fit the structure of the genotypes, we used the criterion *ΔK* [29]:

$$\Delta K=\frac{m\left[\left|L\left(K+1\right)-2L\left(K\right)+L\left(K-1\right)\right|\right]}{\sigma \left[L\left(K\right)\right]}$$ (9)

in which *L(K)* is the average of the natural logarithm of the probability of the data at each step of the MCMC minus half the variance for K populations as estimated by the Structure Harvester app [30].

Given the results of the population structure, the contribution of each hybrid to the population could be determined according to their average value of association probability (*Q*_*ik*_), where *Q*_*ik*_ is the estimated proportion of the genome of the *i-th* genotype derived from the *k-th* ancestral population [28]. From the values of each *Q*_*ik*_ a bar chart of each genotype was built representing the relationship of the *i-th* genotype with the *k-th* ancestral population. Genotypes were considered to be from the same group or population when *Q*_*k*_ exceeded 60%. Genotypes were considered to belong to the mixed population if they did not fit in any population, as previously described [31].

## Results

### Genetic diversity

Of the 768 SNP markers genotyped in the commercial hybrids, 413 SNPs showed minimums of 90% and 5% for call rate and minor allele frequency (MAF), respectively. In the combined population, comprising commercial hybrids and the NAM parents [15], 340 common SNPs were found. Of these, 266 SNPs were selected using the minimums of 90% and 5% call rate and MAF, respectively.

The germplasm of the companies have low estimates of gene diversity (GD), polymorphism information content (PIC), heterozygosity (H) and MAF (Table 3). The one exception was the Syngenta germplasm, which had higher estimates compared to the others, with indices of GD = 0.31, PIC = 0.24, H = 0.38, and MAF = 0.24. In contrast, the lowest index of GD and PIC were found in the germplasm of the Agromen Tecnologia and Santa Helena Sementes companies.

**Table 3:** Number of hybrids (NH) and estimates by company of the gene diversity (GD), polymorphic information content (PIC), heterozygosity (H) and minor allele frequency (MAF).

Company	NH	GD	PIC	H	MAF
Agromen	1	0.04	0.04	0.37	0.04
Biomatrix	3	0.28	0.22	0.31	0.21
Dekalb	4	0.28	0.22	0.34	0.21
Dow Agrosciences	5	0.26	0.21	0.30	0.20
DuPont Pioneer	2	0.27	0.21	0.32	0.22
Santa Helena	1	0.07	0.07	0.31	0.07
Syngenta	4	0.31	0.24	0.38	0.24

Only three of the companies have private alleles, Biomatrix, DuPont Pioneer and Syngenta, with 1, 3, and 11 private alleles, respectively (Table 4). Again, Syngenta hybrids stood out for having the largest number of private alleles, located on chromosomes 1, 4, 5, 6, 7 and 9 (Gramene—www.gramene.org/).

**Table 4:** Private alleles of each germplasm company, chromosome (Chr), chromosome position in kb (Pos), Linked gene, distance to the linked gene in kb (Dist) and ontologies related to the linked gene.

Company	Marker	Chr	Pos (kb)	Linked gene	Dist (kb)	Ontologies
Biomatrix	PZA02970.9	6	98,315.9	GRMZM2G701028	30	-*
D. Pioneer	PZA00065.2	1	49,621.4	GRMZM2G056373	2	-*
	PZA00427.3	6	80,974.9	GRMZM2G103033	90	Protein binding
	PZA02519.7	10	142,184.1	GRMZM2G028902	24	-*
Syngenta	PHM4531.46	1	22,892.8	GRMZM2G312226	Intron	Chitin catabolic process, cell wall macromolecular catabolic process, chitinase activity
	PHM11000.2	1	43,554.6	GRMZM2G030880	Intron	Chloroplast, chloroplast thylakoid membrane
	PZA00975.1	4	9,759.8	GRMZM2G097469	0.04	-*
	PHM14055.6	4	53,441.5	GRMZM2G049525	27	Mitotic cell cycle, DNA recombination
	PHM1307.11	4	56,312.8	GRMZM2G568636	23	Vacuole, oxidation-reduction process
	PZA00401.1	5	56,019.4	GRMZM2G048455	23	MAPK cascade
	PZA03063.18	6	35,896.3	GRMZM2G035741	Intron	-*
	PHM10225.15	7	167,937.9	GRMZM2G150758	8	-*
	PZA03416.7	9	17,000.2	GRMZM2G891465	0.4	-*
	PHM13183.1	9	107,796.8	GRMZM2G055320	13	Catalytic activity
	PHM2278.86	9	112,333.8	GRMZM2G093270	18	-*

Font: Gramene (www.gramene.org/ access at 11^st^ of January of 2015).

* None ontology related until now.

### Genetic vulnerability of Brazilian maize hybrids

#### Effective population size

The within-population inbreeding coefficient (*F*_*st*_) of the 20 hybrids was 0.08, which corresponds to an effective population size (*N*_*e*_) of 18.54 individuals. In contrast, the *N*_*e*_ estimate of the commercial hybrid parents was 127.22 individuals.

#### Correlations between allelic frequencies

The correlations were significant at the 1% level of significance (Table 5). The highest rates of allelic correlation were observed between Dow Agrosciences and Agromen Tecnologia (0.74), Santa Helena Sementes (0.77) and Biomatrix (0.68).

**Table 5:** Correlation between allelic frequencies of 413 SNPs present in the germplasm of the main maize breeding companies in Brazil.

Company	Biomatrix	Dekalb	Dow	Pioneer	SH	Syngenta	Mean
Agromen	0.57**	0.53**	0.74**	0.52**	0.48**	0.47**	0.55
Biomatrix		0.65**	0.68**	0.58**	0.55**	0.53**	0.59
Dekalb			0.60**	0.54**	0.51**	0.54**	0.56
Dow Agrosciences				0.58**	0.77**	0.59**	0.66
DuPont Pioneer					0.44**	0.50**	0.52
Santa Helena (SH)						0.52**	0.54
Syngenta							0.52

** *P* < 0.01 by the t-test.

#### Population structure and genetic diversity of commercial Brazilian hybrids

**Fig 1:**
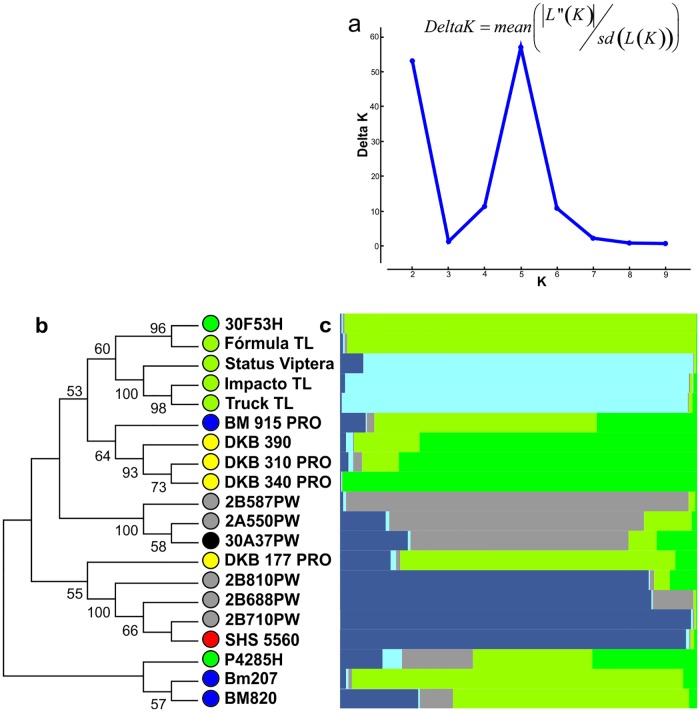
Delta K (Δ*K*) graph obtained by Structure Harvester (a), node consistency dendrogram (b) and population structure of 20 commercial maize hybrids in five populations (c). Colored circles represent the commercial source of the indicated genotype. Agromen Tecnologia—black; Biomatrix—blue; Dekalb—yellow; Dow Agrosciences—gray; DuPont Pioneer—dark green; Santa Helena Sementes—red, and Syngenta—yellowish green.

To generate the population structure of the 20 commercial maize hybrids, we estimated the optimal number of 5 ancestral populations (Fig 1a) according to Δ*K* test [30]. The structure segregated the hybrids into 5 populations (Fig 1c), into which hybrid P4285H (DuPont Pioneer) did not fit and was thus considered a mixture of the five.

The first population comprised 3 Dow Agrosciences hybrids (2B710PW, 2B688PW, 2B810PW) and the Santa Helena Sementes hybrid (SHS 5560). The second population comprised 3 Syngenta hybrids (Truck TL, Impacto TL and Status Viptera). The third population comprised the remaining Dow Agrosciences hybrids (2B587PW, 2A550PW) and the Agromen Tecnologia hybrid (30A37PW). The fourth population comprised the Biomatrix hybrids (BM 207, BM820, BM915 PRO), the last Syngenta hybrid (Fórmula TL), 1 DuPont Pioneer hybrid (30F53H) and 1 Dekalb hybrid (DKB 177 PRO). The fifth population comprised the remaining hybrids from Dekalb (DKB 340 PRO, DKB 310 PRO and DKB 390). Note that of the 5 structured populations, 2 were exclusive to Syngenta and Dekalb.

The dendrogram obtained by bootstrap analysis (Fig 1b) separated these lines into four groups. Group 1 was the most diverse, consisting of 9 hybrids, 4 from Syngenta (Fórmula TL, Status Viptera, Impacto TL, Truck TL), 3 from Dekalb (DKB 390, DKB 310 PRO, DKB 340 PRO), 1 from DuPont Pioneer (30F53H) and 1 from Biomatrix (BM 915 PRO). Group 2 consisted of 2 hybrids from Dow Agrosciences (2B587PW and 2A550PW) and 1 from Agromen Tecnologia (30A37PW). Group 3 consisted of the remaining hybrids from Dow Agrosciences (2B810PW, 2B688PW, 2B710 PW), 1 from Dekalb (DKB 177 PRO) and the Santa Helena Sementes hybrid (SHS 5560). Group 4 included only 2 hybrids from Biomatrix (BM 207 and BM 820). The P4285H hybrid of DuPont Pioneer was not included in any group because it did not have a node consistency greater than 50%.

### Relationships between the Brazilian commercial hybrid germplasms and the parents of the Nested Association Mapping population

The optimal population structure to combine the populations was estimated at K = 2 (S1 Fig). The NAM parents were allocated into only 1 group, except for the B73 inbred line, which was allocated into the same group as the Brazilian hybrids.

**Fig 2:**
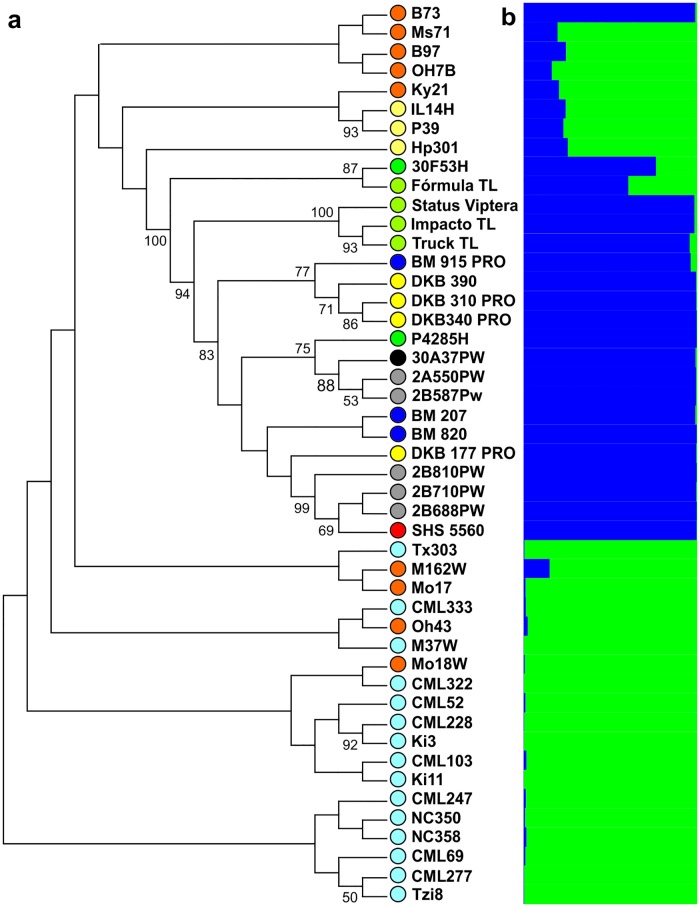
Node consistency dendrogram (a) and population structure of the hybrids and the Nested Association Mapping parents (b). Colored circles represent the company source or origin of the indicated genotype. Agromen Tecnologia—black; Biomatrix—blue; Dekalb—yellow; Dow Agrosciences—gray; DuPont Pioneer—dark green; Santa Helena Sementes—red; Syngenta—yellowish green; tropical inbred lines—ice-blue; temperate inbred lines—orange; and sweet corn or popcorn inbred lines—light yellow.

The dendrogram method separated the genotypes into 7 groups (Fig 2a). Of these, a large group was formed by the Brazilian hybrids, and the other groups were composed of the NAM parents. There was node consistency for both the hybrids and for the NAM parents, however there is a larger clustering among hybrids between the inbred lines. Only the doublet of the parents IL14H with P39, CML228 with Ki3, and CML277 with Tzi8, had node consistencies higher than 50%. The 2 first dendrogram groups obtained by bootstrap have greater similarity to the inbred lines than to the Brazilian hybrids. The hybrids with higher similarity to the NAM parents were the Fórmula TL and 30F53H hybrids.

## Discussion

### Genetic diversity via SNP analysis

The estimates of GD and PIC were low for the companies Agromen Tecnologia and Santa Helena Sementes, due to the low number of hybrids considered for each of these companies. However, the estimates of MAF and H were informative, so there is no predominance of heterozygous loci in these hybrids.

Through the estimates of GD and PIC the genetic diversity of the germplasms and the kinship between hybrids can be inferred. The greater the estimate of these indices, the greater the genetic diversity in the company germplasms and, hence, the lower the kinship between the hybrids of these companies [32]. In this context, Dow Agrosciences had similar estimates to Biomatrix, Dekalb, and DuPont Pioneer, but with more analyzed hybrids. However, the number of hybrids evaluated by company may explain the low estimates of GD and PIC observed in their germplasm.

Syngenta was notably the company with the largest number of private alleles. According to Guo et al. [33], private alleles may infer the genetic diversity of a population. Thus, this high number of private alleles in the Syngenta germplasm indicates that its genetic diversity exceeds that of the other companies.

### Genetic vulnerability of Brazilian maize hybrids

#### Effective population size

The results allow us to infer that the current composition of Brazilian commercial hybrids offers little risk of genetic vulnerability, considering the estimates of genetic variability and *F*_*st*_ between them. According to Sebbenn and Seoane [34], a higher *N*_*e*_, or greater similarity to the actual studied population, occurs when there is negative or close to zero genetic correlation estimates between the genotypes. In this context, the commercial American maize germplasms of the 1980s [18], showed an *N*_*e*_ of 55.9 individuals, corresponding to a *F*_*st*_ of 0.84. This estimate indicates higher genetic vulnerability of these germplasms than that of the current Brazilian commercial germplasms.

However, it is important to emphasize that there are other factors related to genetic vulnerability that were not considered in this study (i.e., the planting area, the market share of each hybrid and the vulnerability to pests and diseases due to resistance genes), suggesting that the low vulnerability found here should be carefully considered.

#### Correlations between allelic frequencies

Through the estimates of allelic frequency correlation, it was observed that there are relationships between the germplasms of the different companies. Of these, the germplasm of Dow Agrosciences has the greatest similarity to other companies. The correlation between Dow Agrosciences and Agromen Tecnologia may be explained by the fact that the latter is affiliated of the former, although they have independent management and production structures. However, there is the possibility of sharing technologies and joint access to the unified germplasm bank (www.dowagro.com/br/agromen/nossa/brasil.htm, accessed January 28, 2015). Despite the high similarity between Dow Agrosciences with Biomatrix and Santa Helena Sementes, there is no known knowledge of any shared germplasms. Nevertheless, it is known that the Helix Sementes owns Biomatrix and Santa Helena Sementes. Santa Helena Sementes was acquired by Helix Sementes in 2011 and its seed stocks, germplasm bank and the commercial structure were transferred to Biomatrix (www.agroceres.com.br/negocios_sementessh.jsp, accessed January 28, 2015). Although these companies are in the same group, they do not have as high of a correlation with each other (0.55) as they do with Dow Agrosciences. This is likely due to the short time between the exchange of material and subsequent development and launch of the evaluated cultivars.

The fact that the levels of allelic correlations were medium to high may be explained by the shared goals of the companies, which seek to achieve genotypes with high adaptability, earlier maturation, resistance to pests and diseases, and greater yields. In addition, many companies had the same source for their original germplasm. Another factor is the obtaining of inbred lines of elite cultivars from other companies, which is permitted by the Brazilian Plant Variety Protection Act. This act makes it clear that the use of protected cultivars for scientific research or as a genetic variability source for plant breeding does not violate the property rights of the protected cultivar.

#### Population structure and genetic diversity of Brazilian commercial hybrids

The use of population structure combined with the dissimilarity dendrograms assisted in differentiation of the genetic groups. The combined use of these methods formed 6 groups with only 3 hybrids not included in any of these groups.

Of the 5 structured populations, 2 are exclusive to a single company. This confirms that there is satisfactory genetic diversity among Brazilian commercial maize hybrids, which is higher between companies than within them. The companies that formed exclusive populations were Dekalb and Syngenta. However, not all hybrids from these companies belong to the same population. For example, in the case of Dow Agrosciences and DuPont Pioneer, there are 2 populations. The grouping of hybrids from same company into 2 or more populations indicates the presence of high genetic variability within the germplasm companies.

Biomatrix has the lowest genetic diversity within their germplasm. This is clearly observed by the grouping of all their hybrids into a single population. The 30A37PW Agromen Tecnologia hybrid showed similarity with hybrids from Dow Agrosciences, confirming the results obtained by the correlation of allele frequencies.

The population structure not only indicated similarity between Dow Agrosciences and Agromen Tecnologia but also clearly indicated the most similar hybrids between them (30A37PW and 2A550PW). This confirms the free access of Agromen Tecnologia to the Dow Agrosciences germplasm bank (www.dowagro.com/br/agromen/nossa/brasil.htm, accessed January 28, 2015). On the other hand, the DKB 177 PRO Dekalb hybrid was the most distinct from the hybrids from the other companies.

As expected with Syngenta having more private alleles, indicating greater genetic diversity within this company compared to the others, the population structure indicated that there is genetic diversity within the Syngenta germplasms. This can be observed by the different groupings of the Fórmula TL hybrid compared to the other hybrids of the Syngenta Company.

Then, the commercial Brazilian maize germplasms have satisfactory GD and N_e_ estimates, with the largest variability between rather than within companies.

### Relationship between the commercial Brazilian hybrid germplasms and the Nested Association Mapping population parents

The results confirm that the NAM parents are genetically divergent from the B73 inbred line [15]. The differences between the B73 and the Mo17 inbred lines, which have been used in breeding programs all around the world to explore heterosis [18], was also evident.

The dendrogram of genetic distances between the commercial hybrids and the NAM parents (Fig 2a) showed that among the NAM parents there is such a high level of genetic variation that there was a low occurrence of node consistency between them. The greatest node consistency rates were observed in the Brazilian hybrids, which form one large group composed of several subgroups. This indicates that the maize breeding in Brazil may have led to a reduction in the germplasm genetic variability. So, in spite of the high estimates of *N*_*e*_, it is necessary to be careful during crop selection to avoid further narrowing the genetic base, as greater index selection for a particular trait can lead to a larger unforeseen loss of genetic variability [35].

It is also clear that there is similarity between the Brazilian commercial hybrids 30F53H and Fórmula TL with some of the parents of temperate origin or for use as Popcorn. This indicates the use of the genotypes of temperate origin in the formation of the current Brazilian commercial germplasm, especially in DuPont Pioneer and Syngenta.

Finally, when analyzing the structure of hybrids with the NAM parents it was evident that the B73 inbred line grouped with the Brazilian hybrids, indicating a coalescence effect. According to Hartl and Clark [36], the coalescence effect is when a population of individuals have a single common ancestor. This coalescence may originate from a founder effect of the B73 inbred line, or its heterotic group of origin (Iowa Stiff Stalk Synthetic), in the Brazilian germplasm. These inbred lines were introduced in Brazil by private companies in the 1990s [37], with the goal of earlier maturation of maize crops.

## Supporting Information

S1 Fig Delta K (ΔK) graph obtained by Structure Harvester. (TIF)
