# Supplementary figures and images for: Genetic Vulnerability and the Relationship of Commercial Germplasms of Maize in Brazil with the Nested Association Mapping Parents
Source: PLoS One. 2016 Oct 25;11(10):e0163739. doi: 10.1371/journal.pone.0163739 (PMC5079593; doi:10.1371/journal.pone.0163739)

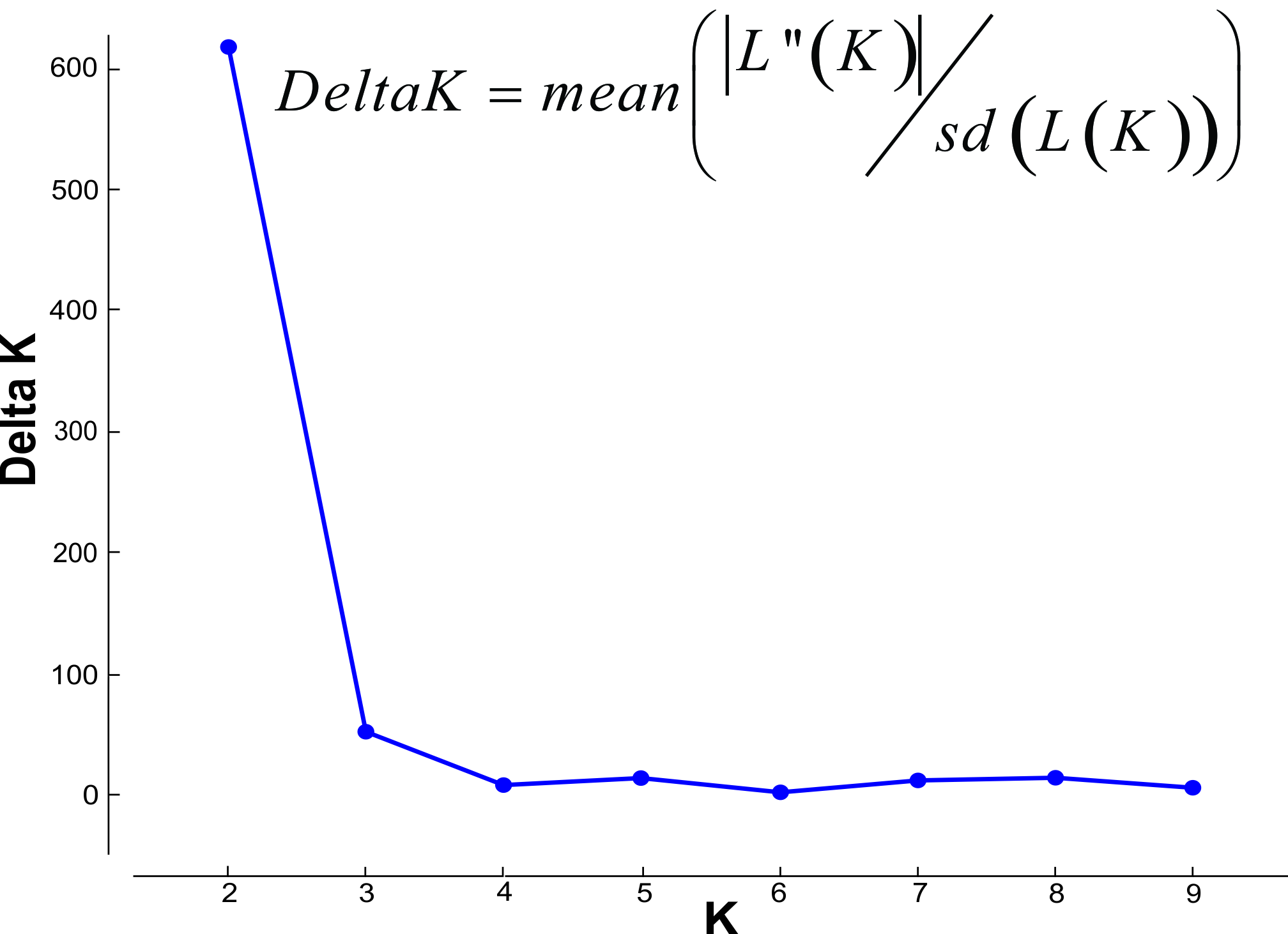

Supplement: S1 Fig — (TIF) [file pone.0163739.s001.tif]
